# Editorial: Silicon and heavy metal stress in plants: current knowledge and future prospects

**DOI:** 10.3389/fpls.2023.1227863

**Published:** 2023-06-12

**Authors:** Zahra Souri, Muhammad Ansar Farooq, Naser Karimi

**Affiliations:** ^1^ Department of Biology, Faculty of Science, Razi University, Kermanshah, Iran; ^2^ Institute of Environmental Sciences and Engineering (IESE), School of Civil and Environmental Engineering (SCEE), National University of Sciences and Technology (NUST), Islamabad, Pakistan

**Keywords:** heavy metal, silicon, detoxification, crops, food security

The indiscriminate discharge and consequent accumulation of heavy metals (HMs) from several anthropogenic sources into the environment is a global concern for crop productivity, food security, and human health. During the last few decades, silicon (Si) has been recognized as one of the beneficial elements involved in ameliorating heavy metal toxicities in plants. The large-scale use of Si fertilizers in field crops is also attracting increasing interest. However, our knowledge about Si and HMs stress in plants is still limited. Therefore, the Research Topic “*Silicon and heavy metal stress in plants: current knowledge and future prospects*” introduces the latest findings in HMs toxicity and the Si-mediated stress tolerance mechanisms in plants so as to promote Si fertilization as a sustainable strategy for mitigating HMs stress and increasing crop productivity. This Research Topic includes four research papers and two review articles.

In the research article by Imran et al., the authors performed the analysis of alternative splicing (AS) under two zinc (Zn) treatment in two different fragrant rice cultivars (Xiangyaxiangzhan and Meixiangzhan-2). RNA-seq analysis revealed dose- and genotype-related AS events in the two rice cultivars. Gene Ontology (GO) analysis showed that these genes were greatly enhanced in a variety of physiological and cellular routes. Moreover, the authors reported that Zn mediates differently the expression of biosynthetic genes of 2-acetyl-1-pyrroline (2-AP). Interestingly, authors also found that genes related to Si, Fe, and other metal-related transport are regulated both at transcriptional and post-transcriptional stages in reaction to Zn. This study highlighted that the biosynthesis pathway of 2-AP and HM transporters can be regulated by epigenetic changes.

In another research article by Rabiya et al., a study was conducted to mediate total chromium (Cr) and lead (Pb) stress in soil and *Solanum melongena* L. This study was done *via* three bacterial species [*Trichococcus* sp. (B1), *Pseudomonas alcaligenes* (B2), and *Bacillus subtilis* (B3)], CP-silica gel (0.2% chitosan polymerized silica gel), and ZnBc (1.5% zinc-enriched biochar). A 24-h biosorption trial revealed that B3 displayed maximum removal efficiency for Cr and Pb. The results of the pot experiment demonstrated that irrigation with Cr- and Pb-contaminated wastewater significantly reduced plant-growth-related parameters. Nevertheless, the application of ZnBc and CP-silica gel treatments in combination with B3 reduced leaf metal accumulation and ultimately improved phyto-tolerance against oxidative stress due to enhanced antioxidant enzyme activities and leaf total phenolic and protein contents. The study concluded that the use of ZnBc and CP-silica gel in combination with B3 could be used as a sustainable approach to minimize the damage caused by total Pb and Cr coming from wastewater.

In a study by Alsamadany et al., the effect of Si-nanoparticle-doped biochar (SBC) was compared to plain biochar (BC) on improving the salinity and arsenic (As) in quinoa (*Chenopodium quinoa*). Results showed that growth, yield, and physiological attributes of quinoa were significantly decreased in the mixed stress of salinity and As, which were improved to variable extents both by BC and SBC application. Overall, the SBC showed to be more effective than BC by reducing As uptake and accumulation, thus reducing the oxidative stress load of plants through the overexpression of antioxidant enzymes. Thus, the authors have proposed the amendment of soil with SBC as an efficacious technique to reduce As contamination and enhance food security.

In another research article, Khan et al. achieved a genome-wide analysis of two transcription factor (TF) families (*WRKY* and *bHLH*) in tomato (*Solanum lycopersicum*) and defined their role against cadmium (Cd) stress. Transcriptomics analysis showed that both genes (*bHLH* and *WRKY*) were highly expressed under Cd stress. Results showed that the most *bHLHs* and *WRKYs* are DNA-binding proteins, which mediate gene expression negatively and positively. The interaction networks analyses showed that these two families are associated with pathways of stress signaling. In *Arabidopsis* genome, the ortholog analysis led to classifying *bHLH* and *WRKY* orthologs into 11 and 9 clusters, respectively, based on their conserved motif compositions, gene structure, and networks of protein–protein interaction. The authors concluded that identifying HM stress-responsive genes (*bHLH* and *WRKY*) in tomato would make available important visions to develop new HM stress-tolerant varieties of tomatoes.

In the review article by Li et al., the authors provided a detailed description of the spectroscopy (optical imaging) coupled with advanced machine learning tools for detecting HMs in plants. Recently, spectroscopy techniques application in HMs detection has gained momentum because of its real-time, *in situ*, and simple action than the traditional chemical analyses techniques. The review summarized the progress of AI-coupled optical imaging spectroscopy techniques application results of plant HM detection over the last decade (2012–2022) and proposed that the challenges of unsatisfactory sensitivity of optimal imaging spectroscopy could be addressed by coupling such analytical and detection methods with advanced AI tools.

In another review article by Nikolić et al., the authors summarized the mechanistic understandings of Si interference under variable conditions of iron (Fe) scarcity and copper (Cu) excess. Fe and Cu are known to share similar transport pathways. Consequently, the inadequate concentration of either of the elements may cause disruptions in the availability and uptake of the other, while Si presence in the growth media affects the distribution of the element supplied above or below optimal levels and the distribution of other microelements and their molar ratios. The effectiveness of Si in the stimulation of metal chelators responsible for the mobilization of deficient elements and excess HMs detoxification has been demonstrated. The authors proposed that an investigation into the mechanisms of silicon’s ameliorative effect is valued for decreasing plant mineral stress and enhancing the nutrient composition of crops.

## Author contributions

All authors listed have made a substantial, direct, and intellectual contribution to the work and approved it for publication.

